# Global, regional, and national burden of spinal cord lesion at neck level a systematic analysis of incidence, prevalence, YLDs with projections to 2046

**DOI:** 10.3389/fpubh.2025.1659091

**Published:** 2025-09-05

**Authors:** Yi Zhang, Weilun Zhao, Xiaoming Peng, Fayun Yang, Shaohui Zong

**Affiliations:** ^1^Department of Spine Osteopathia, The First Affiliated Hospital of Guangxi Medical University, Nanning, China; ^2^Collaborative Innovation Centre of Regenerative Medicine and Medical BioResource Development and Application Co-constructed by the Province and Ministry, Guangxi Medical University, Nanning, China; ^3^Department of Joint and Bone Diseases, Yuebei People’s Hospital, Affiliated Hospital of Shantou University, Shaoguan, China; ^4^Wuming Hospital of Guangxi Medical University, Nanning, China

**Keywords:** spinal cord lesion at neck level, global, disease burden, GBD, 2021

## Abstract

**Background:**

Spinal cord lesion at neck level imposes significant global morbidity, yet cervical-specific burden analysis remains limited.

**Methods:**

Using Global Burden of Disease (GBD) 2021 data (1990–2021), we analyzed incidence, prevalence, and years lived with disability (YLDs) across 204 countries/territories, stratified by sex, age, socio-demographic index (SDI) regions, GBD super regions, and countries. Age-period-cohort (APC) model and Bayesian age-period-cohort (BAPC) model projected trends to 2046.

**Results:**

In 2021, global incidence was 306,568 (age-standardized incidence rate [ASIR] 3.78/100,000), prevalence 7.42 million (age-standardized prevalence rate [ASPR] 88.47/100,000), and YLDs 2.91 million (age-standardized YLDs rate [ASYLDR] 34.72/100,000). Males had higher burdens than females, with cases peaking at 45–64 years. Middle-SDI regions had the highest absolute cases (79,611 incidence), while high-SDI regions showed the highest age-standardized rates (ASRs) (ASIR 5.86/100,000). From 1990–2021, absolute cases rose, but ASRs declined. Projections predict rising absolute cases through 2046.

**Conclusion:**

This study reveals marked regional and demographic disparities in cervical spinal cord lesion burden. Targeted prevention and healthcare planning in high-burden regions are essential to address this global health challenge.

## 1 Introduction

Spinal cord lesion at neck level, a critical neurological disorder, not only disrupts the normal transmission of neural signals but also precipitates a cascade of physiological and psychological challenges, thereby posing a substantial threat to human health (1). Traumatic events, such as motor vehicle accidents, falls, and sports-related injuries, are often the primary culprits behind acute spinal cord lesion at neck level (2). In contrast, non-traumatic causes, including spinal tumors, infectious myelitis, and degenerative spinal diseases, contribute to the chronic forms of these lesions, gradually eroding the quality of life of affected individuals (3). The consequences of spinal cord lesion at neck level can be far-reaching, typically encompassing varying degrees of quadriplegia, respiratory dysfunction due to phrenic nerve involvement, and autonomic dysreflexia, which can lead to potentially life-threatening hypertension spikes (4). Such impairments not only render patients highly dependent on others for daily activities but also impose significant emotional and financial strains on their families, often resulting in a decline in overall family well-being (5).

Over the past few decades, the global burden of spinal cord injuries has been a subject of growing research interest. The Global Burden of Disease (GBD) study, a cornerstone of epidemiological research, has provided a comprehensive overview of the worldwide burden of various diseases and injuries, including spinal cord injuries (6). Through meticulous data synthesis and advanced analytical techniques, the GBD study has enabled researchers and policymakers to identify trends in the incidence, prevalence, and mortality associated with spinal cord injuries on a global scale (7). However, these broad-spectrum analyses often aggregate different types of spinal cord injuries, including those at the neck level, thoracic level, and lumbar level, without offering in-depth insights into the unique burden profile of spinal cord lesion at neck level.

Given the distinct anatomical and physiological characteristics of the cervical spinal cord, spinal cord lesion at neck level typically results in more severe functional impairments compared to lesions at other levels (8). Moreover, the incidence and prevalence of spinal cord lesion at neck level can be significantly influenced by a multitude of factors. For instance, in regions with high-density urban populations and heavy traffic, the risk of traumatic spinal cord lesion at neck level may increase due to a higher incidence of motor vehicle accidents (9). In contrast, in areas with limited access to quality healthcare, non-traumatic spinal cord lesion at neck level may go undiagnosed or untreated, leading to higher long-term disability rates (10). Despite these significant regional and national disparities, current research on the burden of spinal cord lesion at neck level lacks the granularity needed to inform targeted public health interventions.

Furthermore, accurate projections of the future burden of spinal cord lesion at neck level are essential for effective healthcare planning. As populations age globally and the prevalence of risk factors such as obesity and sedentary lifestyles increases, the incidence of non-traumatic spinal cord lesion at neck level due to degenerative diseases is expected to rise (11). Without reliable projections, healthcare systems may struggle to allocate sufficient resources for prevention, treatment, and rehabilitation services, potentially leading to a decline in the quality of care provided to patients with spinal cord lesion at neck level (12).

Against this backdrop, the primary objective of this study is to conduct a comprehensive and systematic analysis of the global, regional, and national burden of spinal cord lesion at neck level. By meticulously examining the incidence, prevalence, and years lived with disability (YLDs) of spinal cord lesion at neck level, and projecting these trends up to 2046, we aim to generate detailed, evidence-based insights. The findings of this study are expected to provide invaluable guidance for healthcare policymakers in formulating targeted prevention strategies, optimizing resource allocation, and enhancing the quality of life for individuals affected by spinal cord lesion at neck level.

## 2 Methods

### 2.1 Data sources

Data for this study were primarily sourced from the GBD 2021 study (13). The GBD study offers a comprehensive, systematic approach to quantifying health loss from diseases, injuries, and risk factors across the globe (14). It integrates data from a vast array of sources, including vital registration systems, hospital admissions, and disease surveillance programs (15). Specifically, we utilized the GBD 2021 database, which contains data on spinal cord lesion at neck level from 1990 to 2021, covering 204 countries and territories, multiple GBD-defined regions, and 5 socio-demographic index (SDI) categories (16). Additionally, the GBD database incorporated data from national and regional health registries for regions where available, aiming to supplement and validate the GBD data (17). For example, some high-income countries maintain detailed spinal cord injury registries that provide granular information on injury causes, patient characteristics, and outcomes (18).

### 2.2 Case definition of spinal cord lesion at neck level

In the GBD 2021 dataset, “spinal cord lesion at neck level” is defined as structural damage or functional impairment of the spinal cord localized to the cervical vertebral level (C1-C7), regardless of etiology. This definition encompasses both complete and incomplete lesions, as well as acute and chronic manifestations, that result in neurological deficits (such as motor, sensory, or autonomic dysfunction) attributable to cervical spinal cord involvement.

Diagnostic identification in the GBD dataset is based on the International Classification of Diseases (ICD) coding systems, including ICD-9-CM and ICD-10. Relevant codes include:

Traumatic cases: ICD-9-CM codes 806 (fracture of cervical vertebra with spinal cord injury), 952.0 (injury to cervical spinal cord); ICD-10 codes S14 (injury to cervical spinal cord), including S14.0 (injury to first cervical spinal cord), S14.1 (injury to second cervical spinal cord), S14.2 (injury to third cervical spinal cord), S14.3 (injury to fourth cervical spinal cord), S14.4 (injury to fifth cervical spinal cord), S14.5 (injury to sixth cervical spinal cord), S14.6 (injury to seventh cervical spinal cord), and S14.9 (injury to cervical spinal cord, unspecified).

Non-traumatic cases: ICD-9-CM codes 336.2 (cervical spondylotic myelopathy), 344.0 (quadriplegia due to non-traumatic spinal cord lesion); ICD-10 codes G95.2 (spinal cord compression, cervical), D33.4 (benign neoplasm of cervical spinal cord), C72.0 (malignant neoplasm of cervical spinal cord), and G04.1 (acute myelitis, cervical).

In the GBD framework, traumatic and non-traumatic cases are systematically distinguished during data abstraction: traumatic cases are categorized under “injuries” in the GBD cause hierarchy, with etiologies linked to external causes (such as motor vehicle accidents, falls, violence), while non-traumatic cases are classified under “non-communicable diseases” or “infectious diseases” (such as tumors, infections, degenerative disorders). For the present study, we extracted data from the GBD 2021 study to comprehensively assess the total burden of spinal cord lesion at neck level.

### 2.3 Study design

This study adopted a retrospective, observational design, leveraging existing data to analyze the burden of spinal cord lesion at neck level. We first characterized the disease burden metrics for 2021 at various levels: global, SDI regions (low, low-middle, middle, high-middle, and high SDI), GBD-defined regions, and individual countries (19). Stratified analyses were performed by sex (male and female) and 5-year age intervals (ranging from 0–4 years to 95 + years) to explore potential variations in disease burden across different demographic subgroups (20). To understand the temporal trends, we analyzed annual data from 1990 to 2021. Hierarchical cluster analysis was then employed to group GBD super regions based on their estimated annual percentage change (EAPC) profiles of disease burden metrics, identifying regions with similar trends in the evolution of the burden of spinal cord lesion at neck level (21). Moreover, we applied the age-period-cohort (APC) model under the maximum likelihood framework and the Bayesian age-period-cohort (BAPC) model to project the disease burden from 2022 to 2046 (22). These approaches inherently assume the continuation of historical trends in age, period, and cohort effects observed during 1990–2021. We acknowledge that unforeseen disruptions, such as large-scale conflicts, pandemics, or abrupt healthcare system reforms, may alter future trajectories beyond what the model can capture. Finally, to address the etiology and prevention potential of neck-level spinal cord lesions, we conducted a systematic decomposition of attributable burden using GBD 2021 risk factor data.

Compared to previous studies, our multi-level and multi-dimensional analysis approach provides a more comprehensive understanding of the burden of spinal cord lesion at neck level. For instance, while some studies have focused on specific regions or age groups (23), our study covers a global scale and analyzes data across all age groups and sexes. Moreover, the use of the APC model and BAPC model for future projections represents an advancement over traditional trend extrapolation methods used in earlier research (24).

### 2.4 Statistical analysis

The initial phase involved a detailed characterization of disease burden metrics for the year 2021. We systematically evaluated three key epidemiological parameters: (1) incidence, (2) prevalence, and (3) YLDs. For each parameter, we assessed both absolute case numbers and age-standardized rates (ASRs) to enable population-adjusted comparisons. These analyses were conducted at multiple hierarchical levels: globally, across SDI (low to high) regions, across GBD-defined regions, and for individual nations. Furthermore, we performed stratified analyses by demographic variables including sex (male/female), 5-year age intervals (0–4 to 95 + years).

Second, to examine longitudinal patterns, we analyzed annual data spanning 1990–2021. Temporal trends were quantified using EAPC metrics, derived from linear regression models applied to log-transformed ASR values. The EAPC provides a standardized measure of the annual change in the rate, allowing for comparisons across different regions and time periods (25). Subsequently, we implemented hierarchical cluster analysis to classify GBD super regions into distinct trajectory groups based on their EAPC profiles, thereby identifying geographical clusters exhibiting similar temporal patterns in disease burden evolution.

Moreover, to predict the future disease burden from 2022 to 2046, the APC under the maximum likelihood framework and the BAPC model were applied. The APC model and BAPC model is a powerful tool for disentangling the effects of age, time period, and birth cohort on disease incidence or prevalence. It allows for a more accurate prediction of future disease trends by taking into account these different factors (26). By estimating the age, period, and cohort effects simultaneously, the APC model and BAPC model can capture complex patterns of disease occurrence that may be missed by simpler models (27).

Finally, the burden of attribution is decomposed systematically and the risk factors are analyzed.

Statistical significance was determined when the *p*-value was less than 0.05. All statistical analyses, including database construction, collation, and analysis, were performed using the R (version 4.0.2) software. We utilized various R packages, such as “dplyr” for data manipulation and “ggplot2” for data visualization (28).

## 3 Results

### 3.1 Disease burden of neck-level spinal cord lesions in 2021

In 2021, the global incidence of neck-level spinal cord lesions was 306,568 cases [95% uncertainty intervals (UI): 216,989-456,717], with an age-standardized incidence rate (ASIR) of 3.78 per 100,000 population (95% UI: 2.69–5.60). Prevalence reached 7.42 million cases (95% UI: 6,744,305-8,352,639), corresponding to an age-standardized prevalence rate (ASPR) of 88.47 per 100,000 (95% UI: 80.41–99.90). YLDs totaled 2.91 million (95% UI: 2,070,218-3,764,134), with an age-standardized YLDs rate (ASYLDR) of 34.72 per 100,000 (95% UI: 24.77–44.95) (Supplementary Tables 1–3).

Males exhibited approximately 30% higher burden than females across incidence, prevalence, YLDs, and their respective ASRs (Supplementary Figure S1; Supplementary Tables 1–3).

Age-specific distribution (Supplementary Figure S2) showed that both case counts and ASRs increased with age initially, peaking in the 45–64 years group, followed by a gradual decline (Supplementary Figure S2; Supplementary Tables 1–3).

At the SDI regional level, the middle SDI region had the highest absolute counts: 79,611 incident cases (95% UI: 55,116-119,153), 2,048,787 prevalent cases (95% UI: 1,845,151-2,365,385), and 812,670 YLDs (95% UI: 580,634-1,065,285). In contrast, the low SDI region had the lowest absolute burden: 35,216 incident cases (95% UI: 23,544-56,669), 601,907 prevalent cases (95% UI: 451,373-880,208), and 265,014 YLDs (95% UI: 171,368-421,315). For ASRs, high SDI regions ranked highest (ASIR: 5.86, 95% UI: 4.00–8.87; ASPR: 141.92, 95% UI: 130.44–154.92; ASYLDR: 53.03, 95% UI: 37.82–68.37), while low-middle SDI regions had the lowest (ASIR: 2.92, 95% UI: 2.05–4.37; ASPR: 59.15, 95% UI: 53.28–66.74; ASYLDR: 24.68, 95% UI: 17.80–31.91; Supplementary Figure S3; Supplementary Tables 1–3).

Based on the comprehensive GBD 2021 data, substantial disparities in the burden of spinal cord lesions at the neck level were observed across super regions. High-income regions bore the highest absolute burden, accounting for 80,639 incident cases (26.4% of the global total; 95% UI: 52,358-131,147), 2,189,184 prevalent cases (95% UI: 2,004,303-2,412,320), and 804,438 YLDs (95% UI: 581,439-1,035,355). In contrast, the Gulf Cooperation Council had the lowest incidence (4,256 cases; 95% UI: 2,988-6,283) and prevalence (97,686 cases; 95% UI: 88,577-109,380), while the Nordic Region recorded the fewest YLDs (21,304; 95% UI: 15,214-27,921). ASRs further highlighted these disparities: The European Union had the highest incidence rate (6.26/100,000; 95% UI: 4.18–9.91), while Sub-Saharan Africa had the lowest (2.34/100,000; 95% UI: 1.75–3.21). For prevalence and YLDs rates, the Nordic Region ranked highest (164.26/100,000 prevalence; 95% UI: 147.37–185.07 and 61.82/100,000 YLDs; 95% UI: 43.68–81.74), whereas Sub-Saharan Africa again showed the lowest prevalence rate (49.31/100,000; 95% UI: 39.65–66.06) and Sahel Region the lowest YLDs rate (24.02/100,000; 95% UI: 16.50–35.61; Supplementary Tables 1–3; Supplementary Figure S4).

National-level variations were substantial. For example, Afghanistan had the highest incident cases (7,028; 95% UI: 2,939-14,993) and ASIR (20.23 per 100,000; 95% UI: 8.79–43.46) among selected countries, while American Samoa had the lowest (1 incident case; 95% UI: 1–2; ASIR: 2.19 per 100,000; 95% UI: 1.54–3.31). Similar patterns were observed for prevalence and YLDs: Afghanistan had the highest prevalent cases (79,383; 95% UI: 35,962-166,898), ASPR (326.1 per 100,000; 95% UI: 139.06–702.12), YLDs (34,980; 95% UI: 15,109-73,751), and ASYLDR (140.49 per 100,000; 95% UI: 61.37–299.21), while Algeria had the lowest ASIR (2.85 per 100,000) and ASPR (82.41 per 100,000) among the same group (Supplementary Table 1).

### 3.2 Temporal trends in neck-level spinal cord lesion burden, 1990–2021

Globally, absolute counts of incident, prevalent, and YLDs cases increased from 1990 to 2021: incidence rose from 249,122 (95% UI: 183,942-345,205) to 306,568, prevalence from 5,212,979 (95% UI: 4,765,776-5,759,968) to 7,423,601, and YLDs from 2,152,418 (95% UI: 1,539,248-2,733,084) to 2,905,920. Conversely, s declined: ASIR decreased from 4.88 (95% UI: 3.56–6.88) to 3.78, ASPR from 107.05 (95% UI: 98.39–116.87) to 88.47, and ASYLDR from 43.8 (95% UI: 31.45–55.38) to 34.72 per 100,000 (Figure 2; Supplementary Tables 1–3).

**Figure 1:**
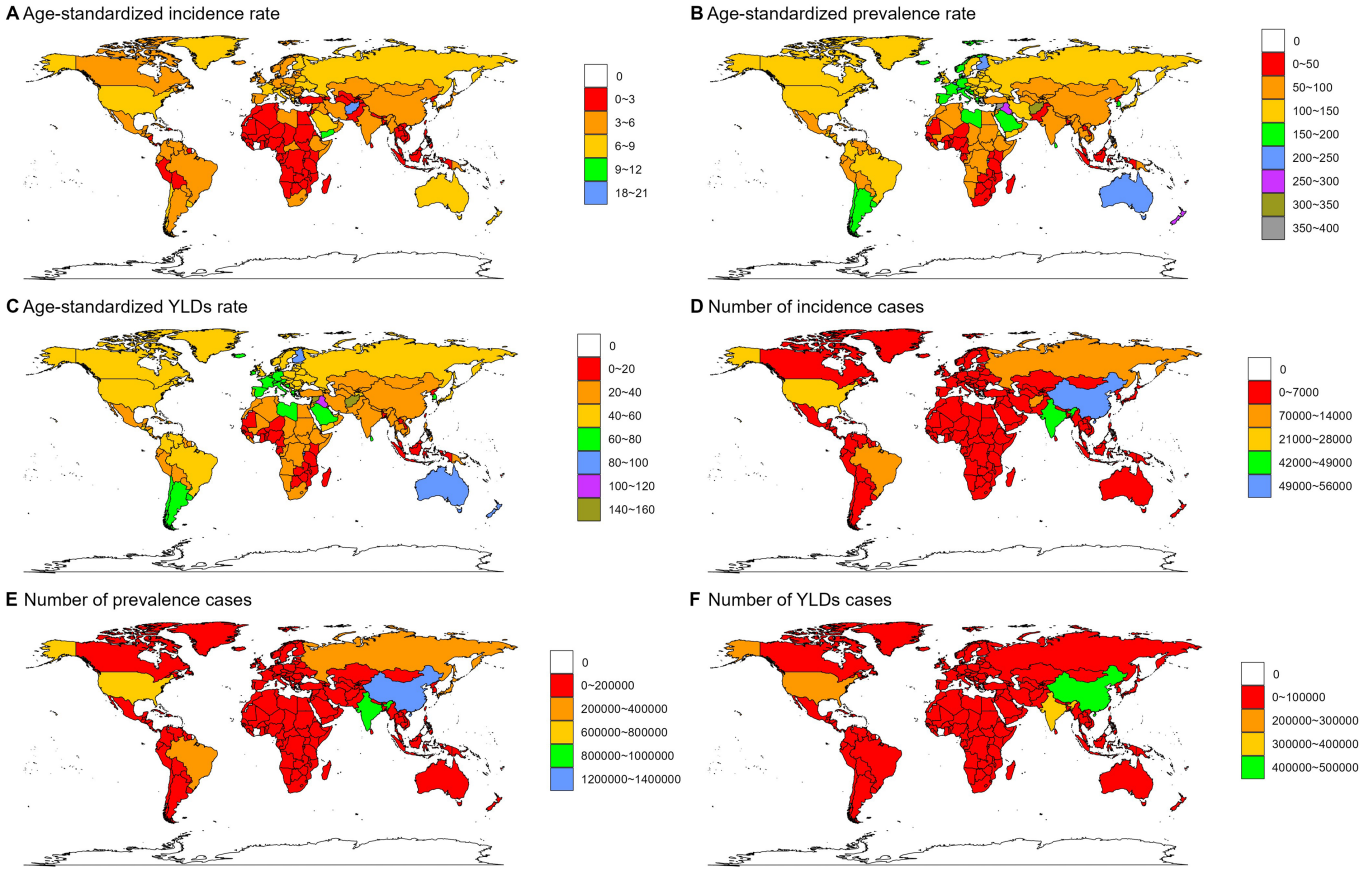
Numbers and age-standardized rates of spinal cord lesion at neck level-related incidence, prevalence, YLDs across countries and territories in 2021.

**Figure 2:**
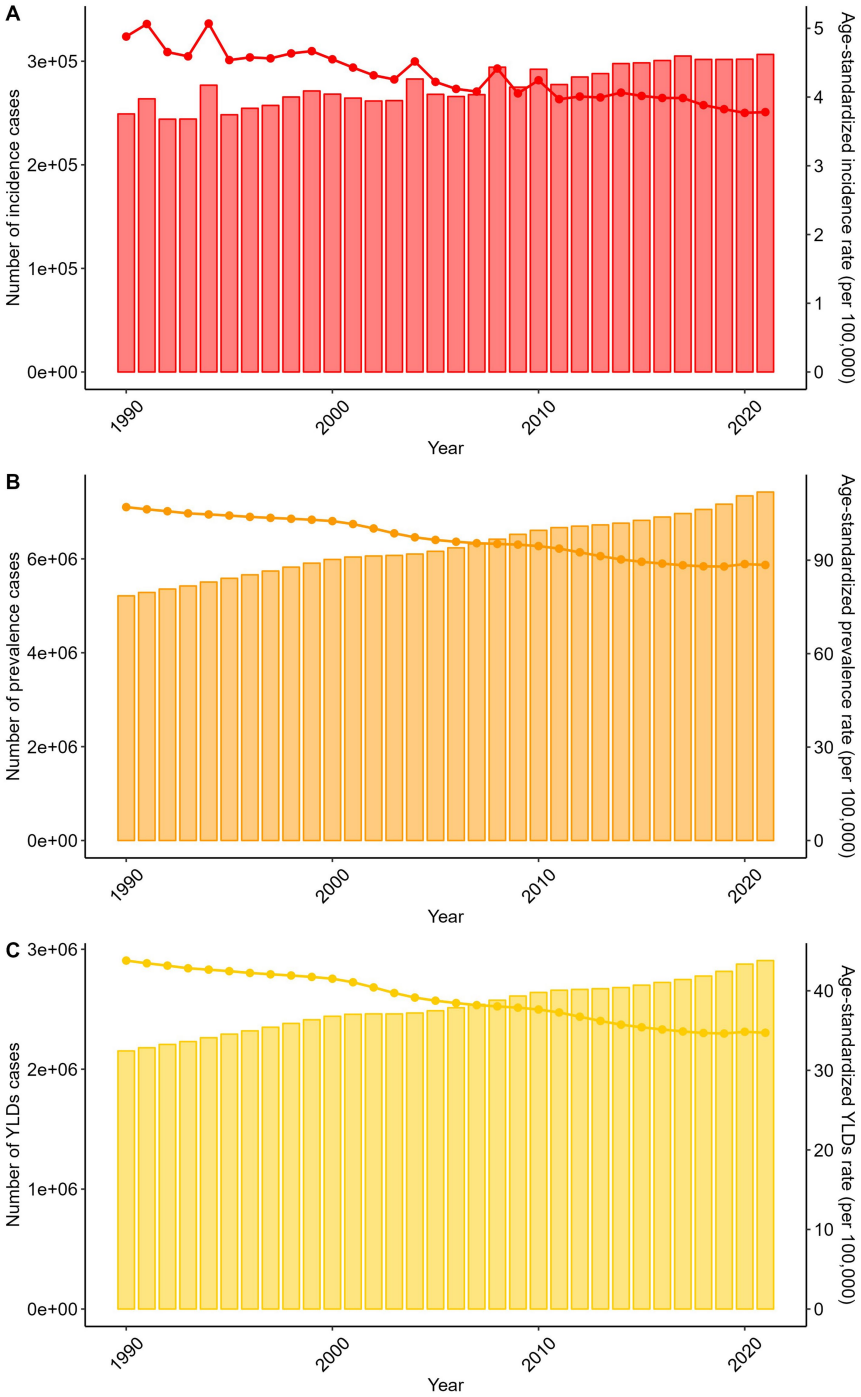
Trends in the numbers and age-standardized rates of spinal cord lesion at neck level-related incidence, prevalence, YLDs globally from 1990 to 2021.

**Figure 3:**
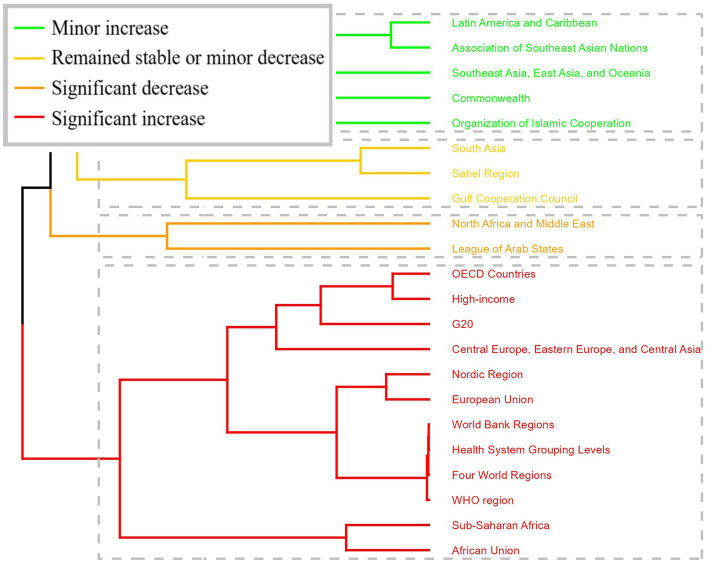
Results of cluster analysis based on the EAPC values of the spinal cord lesion at neck level-related age-standardized rates for incidence, prevalence, YLDs from 1990 to 2021.

**Figure 4:**
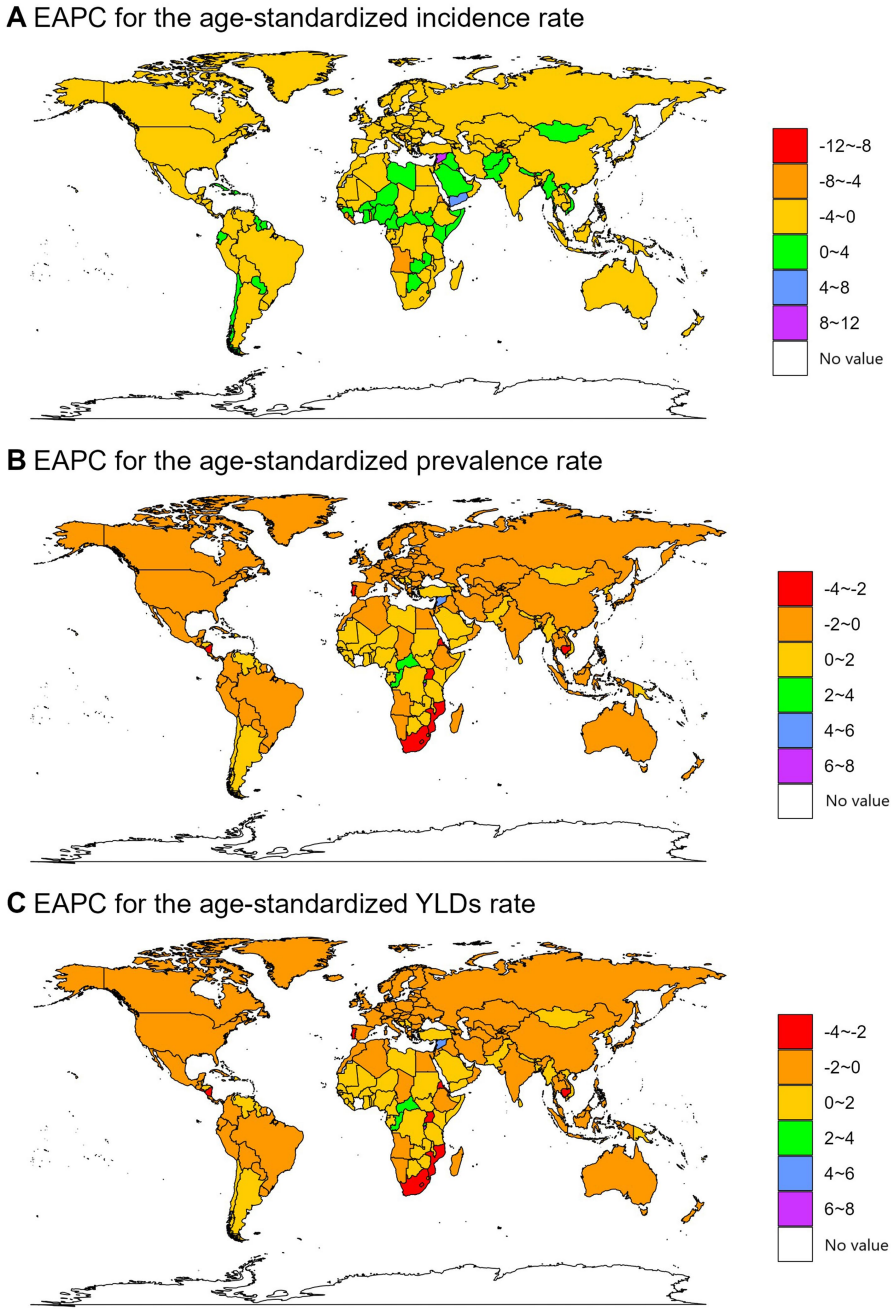
The EAPC of spinal cord lesion at neck level-related ASRs from 1990 to 2021.

**Figure 5:**
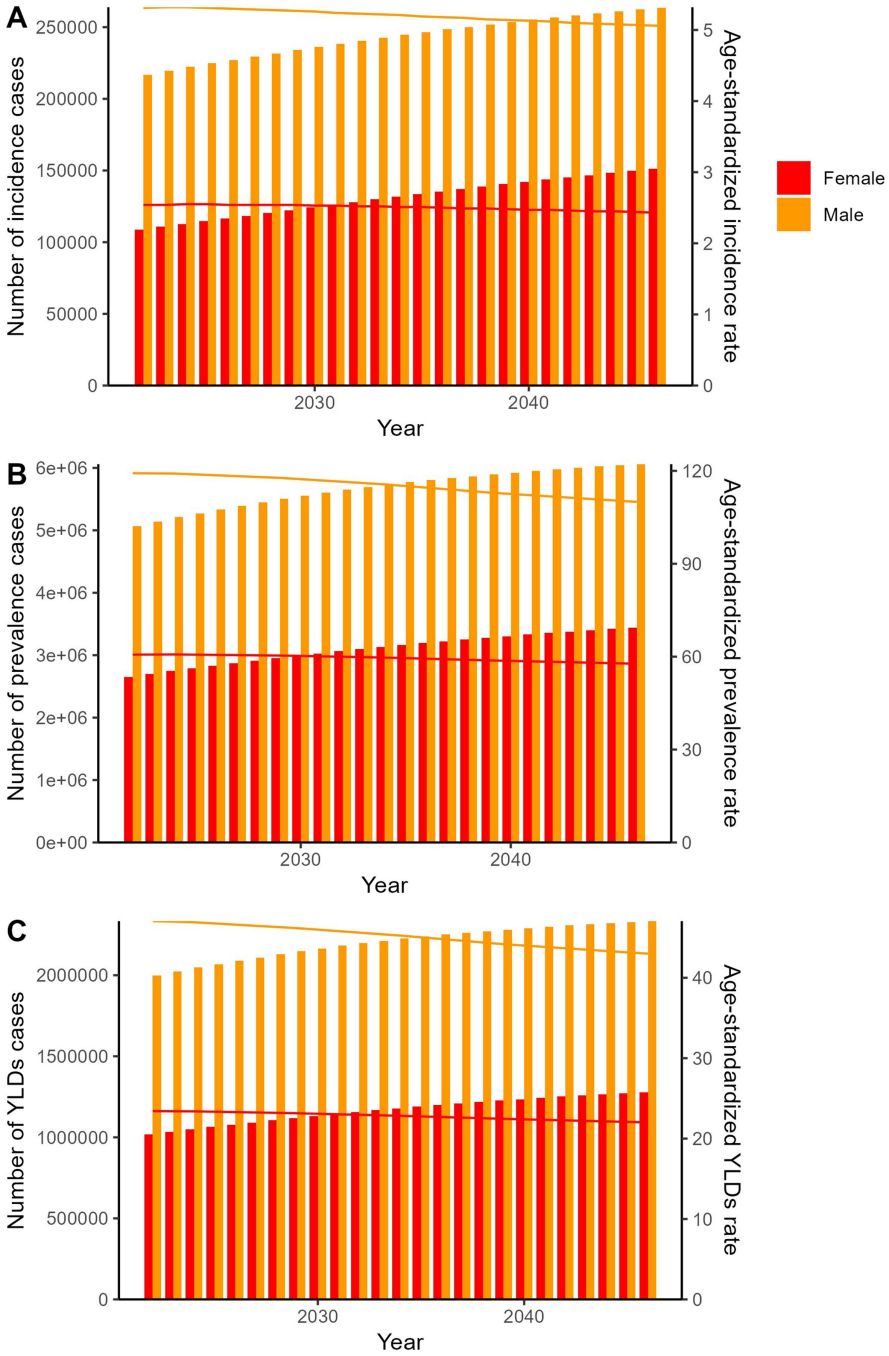
The predicted results in the spinal cord lesion at neck level-related numbers and age-standardized rates of incidence, prevalence, YLDs by sex globally from 2022 to 2046 of the APC model.

**Figure 6:**
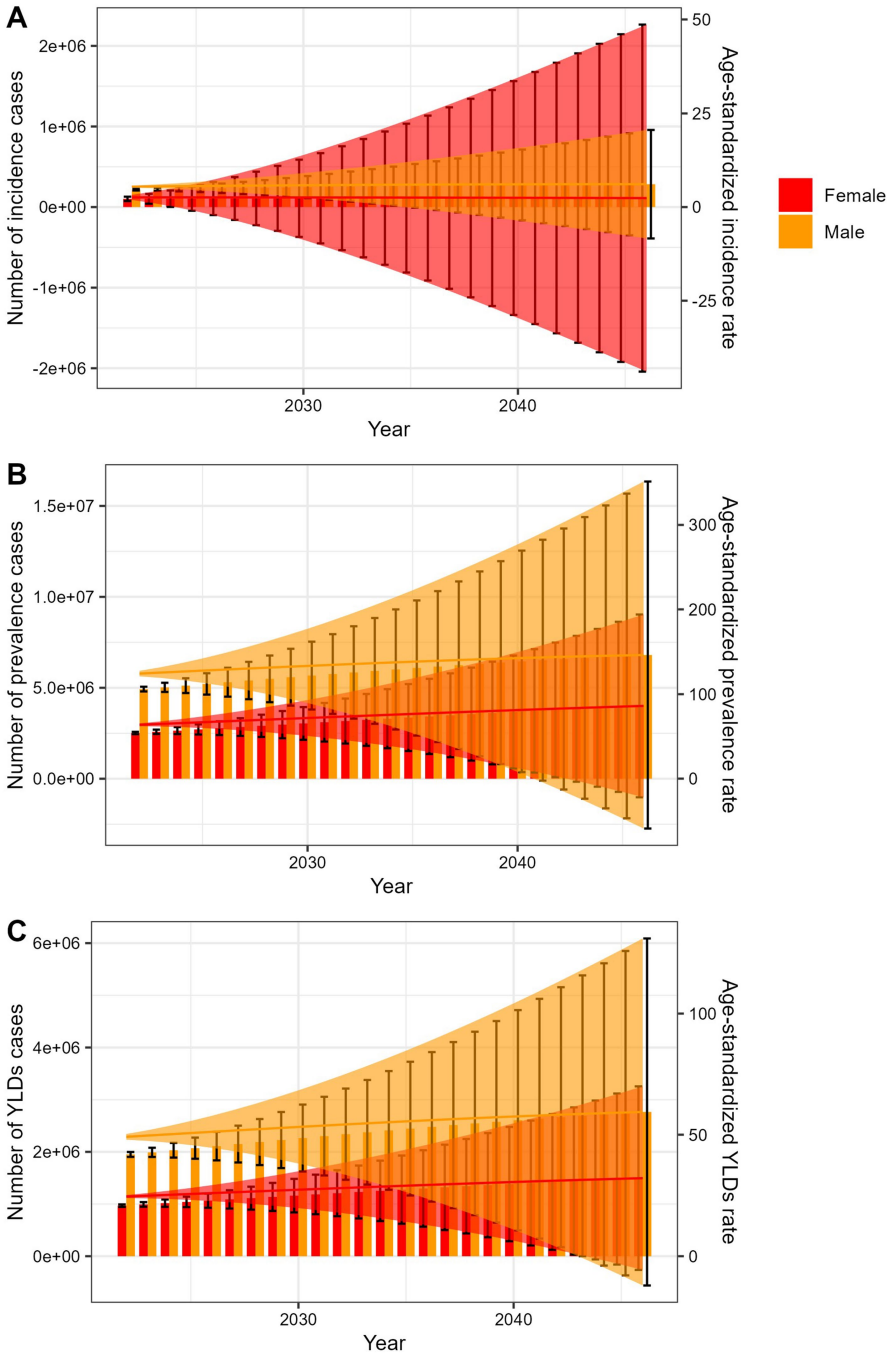
The predicted results in the spinal cord lesion at neck level-related numbers and age-standardized rates of incidence, prevalence, YLDs by sex globally from 2022 to 2046 of the BAPC model.

Trends were consistent across sexes (Supplementary Figure S5; Supplementary Tables 1–3), younger age groups (Supplementary Figure S6; Supplementary Tables 1–3), and all SDI regions (Supplementary Figure S7; Supplementary Tables 1–3).

Cluster analysis of GBD super regions (Figure 3) identified distinct trajectory patterns: OECD Countries, High-income, G2O, Central Europe, Eastern Europe, and Central Asia, Nordic Region, European Union, World Bank Regions, Health System Grouping Levels, Four World Regions, WHO region, Sub-Saharan Africa, African Union showed significant increases in incidence, prevalence, and YLDs rates, while regions including North Africa and Middle East and League of Arab States exhibited significant decreases (Figure 3).

National trends also varied. Yemen had the largest increase in ASIR [estimated annual percentage change (EAPC) = 4.70, 95% confidence interval (CI): 3.28–6.14], while Burundi had the largest increases in ASPR (EAPC = 6.91, 95% CI: 4.60–9.28) and ASYLDR (EAPC = 6.69, 95% CI: 4.37–9.07). Timor-Leste showed the largest decrease in ASIR (EAPC = −8.03, 95% CI: −10.44 to −5.56), while Mozambique had the largest decreases in ASPR (EAPC = −3.98, 95% CI: −4.16 to −3.80) and ASYLDR (EAPC = −4.10, 95% CI: −4.26 to −3.93; Figure 4; Supplementary Tables 1–3).

### 3.3 Projections, 2022–2046

The APC model projected increases in absolute incidence, prevalence, and YLDs counts from 2022 to 2046, alongside continued declines in ASRs for both sexes (Figure 5; Supplementary Table 4). The BAPC model showed that the number of cases and ASRs would all increasing in the next 25 years (Figure 6; Supplementary Table 5).

### 3.4 Risk factor analysis for spinal cord lesions at neck level

Our analysis focused on the proportion of disease burden attributed to different risk factors for spinal cord lesions at the neck level across various locations and years. In the high-middle SDI region in 1990, poisoning by carbon monoxide accounted for 0.1% of the ASIR, number of incidence cases, ASPR, number of prevalence cases, ASYLDR, and number of YLDs cases. This suggests a relatively minor contribution of this risk factor to the overall burden in this area and year. Non-venomous animal contact had an ASIR proportion of 1.0 and 0.9% in terms of the number of incidence cases, while prevalence and YLDs related proportions were 0.0%. It implies that this factor had a limited role in prevalence and YLDs despite some impact on incidence. Unintentional firearm injuries showed more significant contributions. It accounted for 1.1% of both the ASIR and number of incidence cases, 1.3% of the ASPR and number of prevalence cases, and 1.3% of the ASYLDR and number of YLDs cases. This indicates a relatively substantial role in causing the burden of neck-level spinal cord lesions. Venomous animal contact had negligible contributions with 0.1% in incidence-related proportions and 0.0% in prevalence and YLDs proportions. Conflict and terrorism contributed 0.9% in most metrics except for the number of YLDs cases where it was 1.0%, highlighting its influence on the burden, especially in terms of YLDs (Supplementary Figure S8; Supplementary Table 6).

## 4 Discussion

This study provides a comprehensive analysis of the global, regional, and national burden of neck-level spinal cord lesions, including incidence, prevalence, and YLDs, with projections through 2046. In 2021, the global burden of neck-level spinal cord lesions was substantial, with marked variations across demographic subgroups, SDI regions, GBD super regions, and countries. Males consistently exhibited a higher burden than females, and the burden peaked in the 45–64 age group before declining. The middle SDI region had the highest absolute case counts, while high SDI regions showed the highest ASRs. Regionally, Asia carried the greatest absolute burden across all metrics, whereas Oceania had the lowest. Notably, Australasia displayed extremely high ASRs, indicating a severe per-capita impact (17).

The observed gender disparities align with prior research on spinal cord injuries. Males are more likely to engage in high-risk activities (such as certain sports, manual labor), increasing their susceptibility to traumatic lesions (29). Additionally, occupational exposures may elevate their risk of non-traumatic causes, such as degenerative spinal conditions (30). These findings underscore the need for gender-tailored prevention and intervention strategies.

The age-related burden pattern reflects the natural history of neck-level spinal cord lesions. Younger individuals face higher risks of traumatic injuries due to greater participation in physical activities and accident exposure (31). With aging, non-traumatic causes (such as spinal stenosis, tumors) become more prevalent, driving increases in prevalence and YLDs. The subsequent decline in burden among older age groups may stem from shorter life expectancies in severely affected individuals or underdiagnosis in this population (32).

Regional disparities in burden are shaped by multiple factors. High-income regions often report higher ASRs, likely due to superior diagnostic capabilities and more robust reporting systems (33). In contrast, low-income regions exhibit lower rates, which may not reflect true burden, limited healthcare access, inadequate diagnostic infrastructure, and poor surveillance could lead to underreporting (34). Lifestyle differences, environmental factors, and varying risk factor prevalence further contribute to regional variations (35).

We wish to critically address the observed near-zero estimates in certain regions. These figures likely reflect substantial data gaps rather than true disease absence. In low-income settings, three key factors may contribute to systematic underreporting. Firstly, limited diagnostic capabilities for non-traumatic lesions (such as cervical spondylotic myelopathy often misdiagnosed as arthritis). Moreover, incomplete trauma registries in conflict zones. Finally, lack of community-based surveillance for mild impairments (13). Consequently, our reported estimates for these regions should be interpreted as minimum probable burdens.

Building on the observed disparities in YLDs burden across SDI levels, our findings carry significant economic implications for policy planning. The stark contrast between high-SDI regions (highest ASYLDR) and low-SDI regions (lowest absolute YLDs but greatest healthcare access barriers) suggests fundamentally different intervention priorities. In resource-limited settings, where our data show higher traumatic injury rates (such as Afghanistan), investing in cost-effective primary prevention, such as road safety programs and fall prevention, may yield the highest return by reducing incidence. Conversely, in high-SDI regions with aging populations (such as Australasia), the focus should shift to optimizing rehabilitation access and long-term care systems to reduce disability severity. Critically, without region-specific economic analyses of interventions (beyond this study’s scope), the observed burden heterogeneity underscores that a “one-size-fits-all” approach would be both clinically ineffective and economically inefficient.

Temporal trends from 1990 to 2021 show global increases in absolute case counts but declines in ASRs. This pattern likely reflects population growth and aging (driving absolute increases) alongside improvements in healthcare, injury prevention, and public awareness (contributing to reduced ASRs) (4). Regional variations in trends are notable: increases in Oceania, the Caribbean, and Central Africa may relate to demographic shifts, rising risk factors, or improved data collection (36), while declines in high-income regions likely stem from more effective prevention, treatment, and rehabilitation (24).

Projections for 2022–2046 suggest rising absolute incidence, prevalence, and YLDs counts, with continued declines in ASRs. These trends indicate that while population growth and aging will expand the overall burden, per-capita burden may decrease if current progress in prevention, treatment, and healthcare persists (37). However, projections are subject to uncertainties, including changes in risk factors, new treatment modalities, and policy shifts (38).

Our results on the proportion of disease burden from various risk factors for neck-level spinal cord lesions reveal important patterns. Carbon monoxide poisoning’s minor contribution may signal effective preventive measures in the high-middle SDI region in 1990. These could include ventilation regulations and awareness campaigns, which other regions might adopt. Non-venomous animal contact’s impact on incidence but not prevalence or YLDs implies mild initial injuries. Still, better first-aid and early intervention could further reduce any long-term effects. The substantial role of unintentional firearm injuries is alarming. Stricter gun control, safety training, and public awareness are urgently needed to lower the burden they cause. Venomous animal contact’s negligible impact on prevalence and YLDs likely stems from accessible antivenom and trained medical staff. This success can be replicated elsewhere. Conflict and terrorism’s higher contribution to YLDs shows severe, long-lasting disabilities from such events. Post-conflict and post-terrorism rehabilitation services must be enhanced through international and local cooperation. In conclusion, these findings offer a basis for targeted strategies. Future research should extend this analysis globally to create more comprehensive prevention plans.

Our findings, derived from the Global Burden of Disease (GBD) study, should be interpreted in light of the methodological framework underpinning its non-fatal data. Most non-fatal estimates (such as prevalence, incidence, and years lived with disability) in the GBD database are statistically modeled outputs rather than directly observed measurements. To generate globally comparable estimates across >200 countries and territories for nearly all diseases and injuries, the GBD study employs advanced Bayesian meta-regression tools (such as DisMod-MR) and complex hierarchical models. These integrate sparse, heterogeneous, and often fragmented raw data sources (such as national surveys, hospital registries, epidemiological publications) while adjusting for biases and gaps in data availability. This model-dependent approach inherently carries assumptions about data missingness mechanisms, temporal and spatial trend transferability, covariate relationships, and prior distributions. While the GBD consortium rigorously quantifies known uncertainties (such as via 1,000 draw-based uncertainty intervals propagating sampling error and data variability), violations of core model assumptions may introduce unknown uncertainties that are challenging to fully characterize or quantify. For instance, if disease progression parameters or healthcare access biases differ substantially from model priors in specific subpopulations, or if unmeasured confounders exist in regions with extremely limited data, estimates may deviate from true values in ways not captured by conventional uncertainty intervals. Consequently, we emphasize that GBD estimates represent the best available synthesized evidence for global health prioritization but are not equivalent to gold-standard, universally validated measurements. The GBD team continuously refines modeling frameworks and incorporates new data sources to mitigate these concerns; nevertheless, users must remain cautious regarding structural uncertainties arising from the modeling process itself.

This study has several limitations. First, reliance on the GBD database, despite its comprehensiveness, introduces potential inaccuracies or biases, particularly in regions with limited data collection capacity (39). Second, we acknowledge that, due to data availability constraints, the current study aggregates all causes of neck-level spinal cord lesions and is unable to disaggregate the burden by specific etiologies (such as traumatic and degenerative disease). This limitation may restrict the applicability of our findings in guiding etiology-specific prevention and management strategies, as different etiologies often require distinct intervention approaches (40). Third, the APC projections assume continuity of historical trends, an inherent limitation of this methodology. Global disruptions could substantially deviate future burden patterns from our estimates. For example, in conflict-affected regions, war-related trauma surges may accelerate incidence trends. Pandemics could divert healthcare resources from spinal care, worsening disability severity. Demographic shocks (such as mass migration) may alter regional age structures. Such events highlight the need for dynamic surveillance models beyond APC frameworks (41). Fourth, near-zero estimates in select regions require cautious interpretation. While GBD’s Bayesian modeling mitigates data gaps through cross-country borrowing, fundamental surveillance limitations in fragile states may result in underestimation. These figures more likely indicate health information system fragilities than true epidemiological patterns. Future studies should incorporate ground-level validation studies where feasible. Finally, technical limitations in our predictive model’s (APC model) calculation of transmission uncertainty prevent the inclusion of a 95% CI in the 2046 projection. Future iterations will prioritize methodological improvements to incorporate uncertainty bands into long-term projections, aligning with the best practices of the GBD.

## 5 Conclusion

In conclusion, this study provides valuable insights into the global, regional, and national burden of spinal cord lesion at neck level, its temporal trends, and future projections. The findings highlight the need for targeted public health interventions, especially in regions with a high burden and among vulnerable populations. Future research should aim to address the limitations of this study, such as improving data quality, exploring specific causes, and refining prediction models, to better inform healthcare planning and resource allocation for the management of spinal cord lesion at neck level.

## Data Availability

The original contributions presented in the study are included in the article/Supplementary material, further inquiries can be directed to the corresponding author/s.
