# Supplementary figures and images for: Global, regional, and national burden of spinal cord lesion at neck level a systematic analysis of incidence, prevalence, YLDs with projections to 2046
Source: Front Public Health. 2025 Sep 5;13:1659091. doi: 10.3389/fpubh.2025.1659091 (PMC12446318; doi:10.3389/fpubh.2025.1659091)

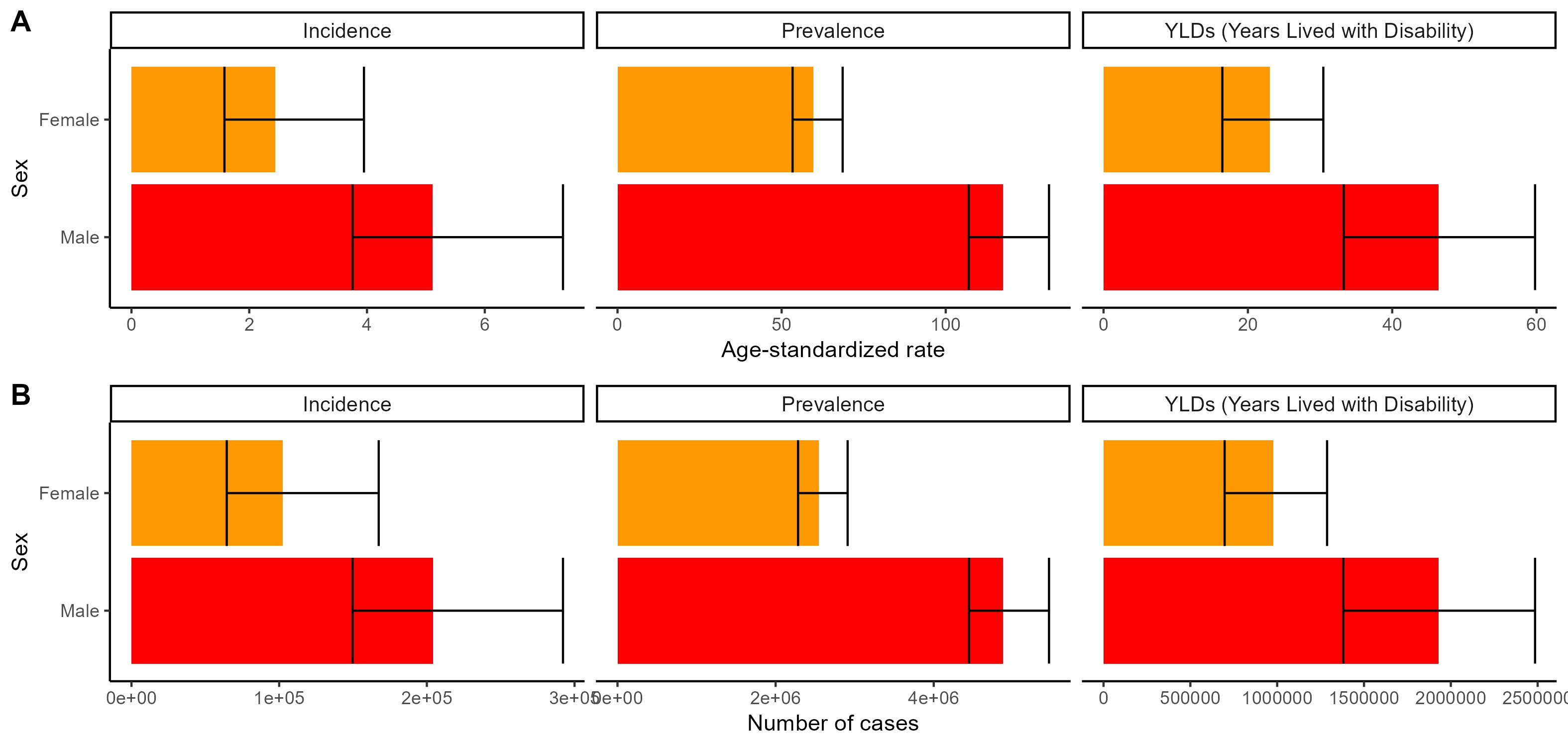

Supplement: Supplementary file 7 [file Figure_1.JPEG]

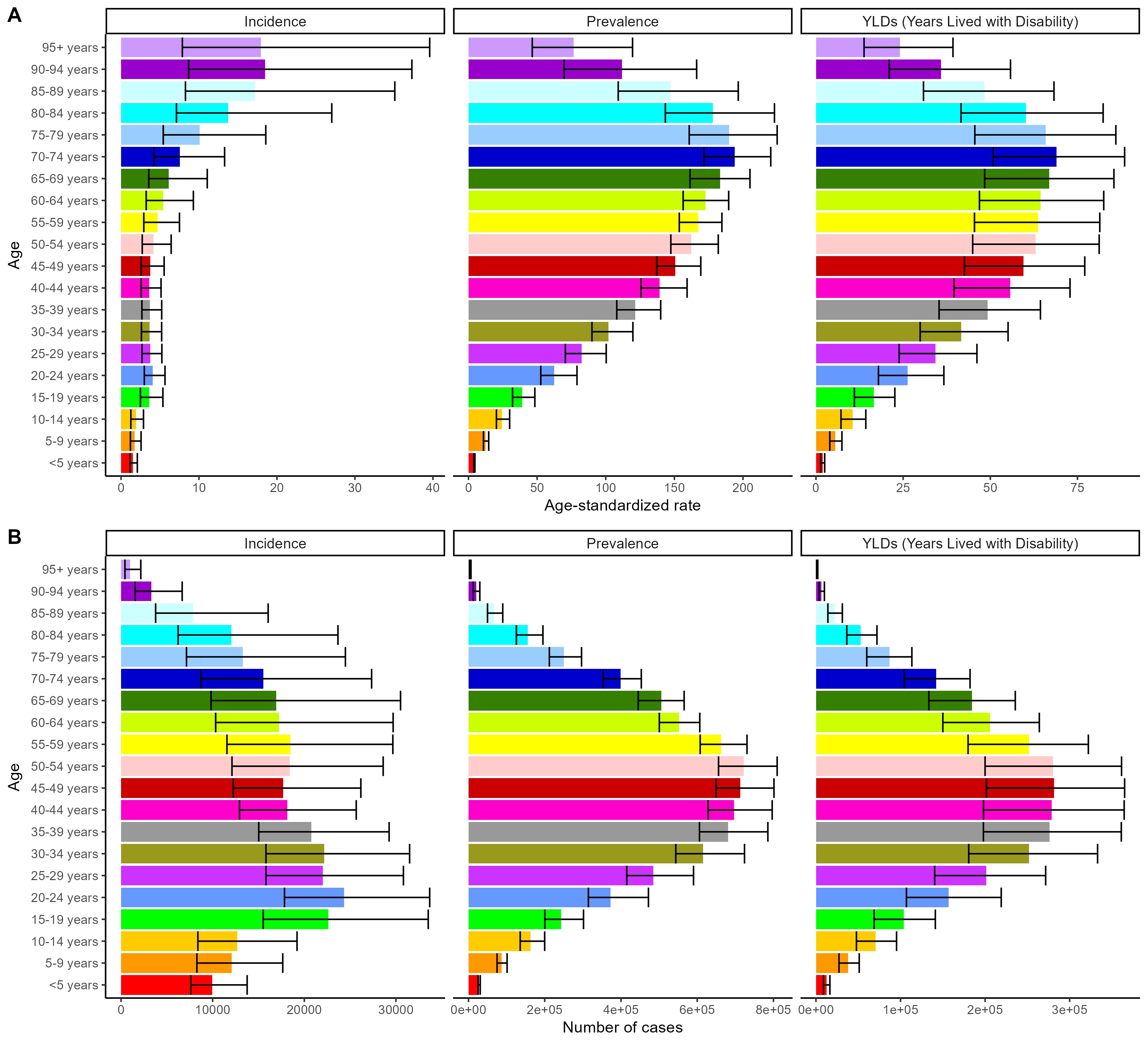

Supplement: Supplementary file 8 [file Figure_2.JPEG]

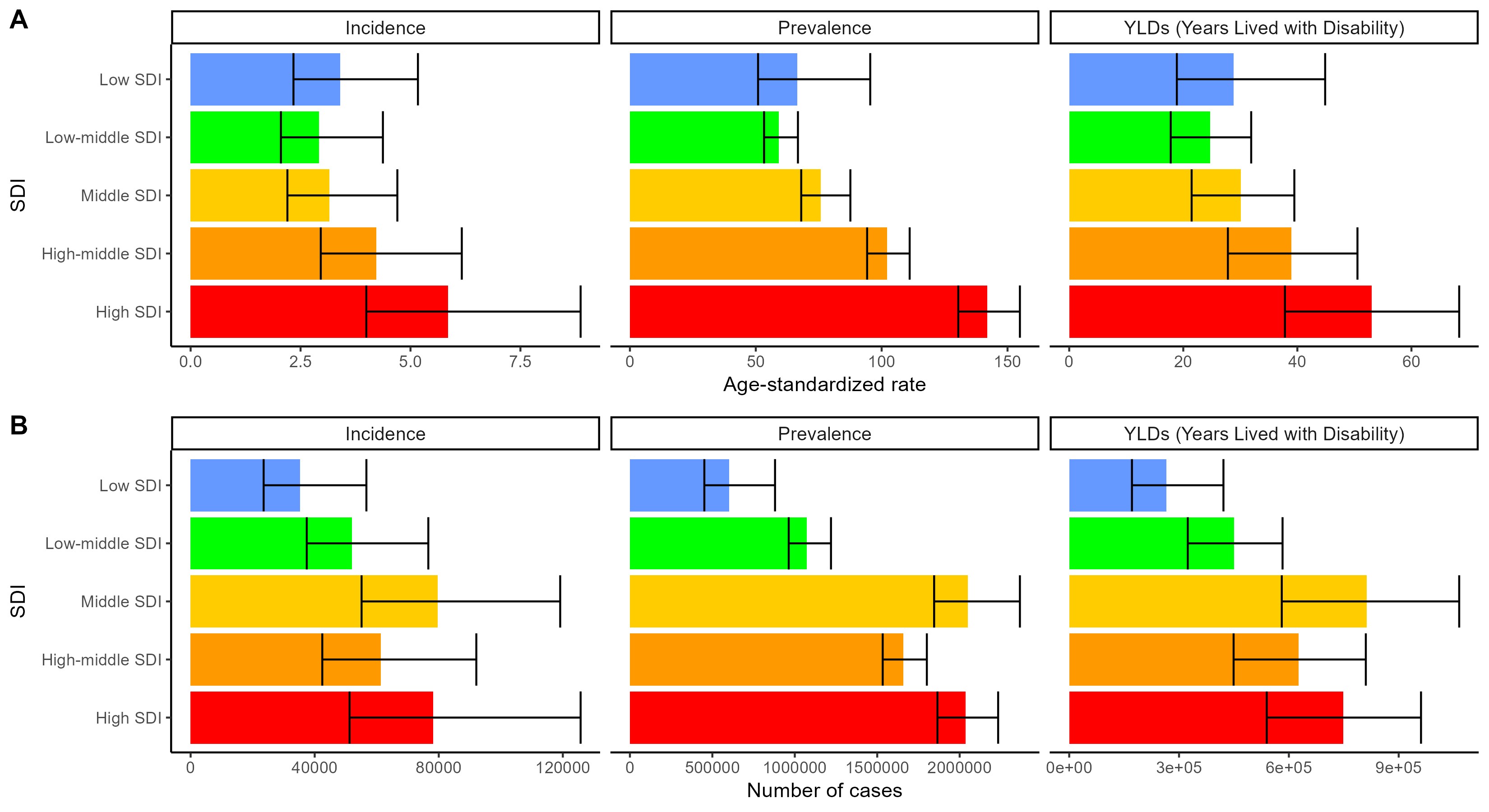

Supplement: Supplementary file 9 [file Figure_3.JPEG]

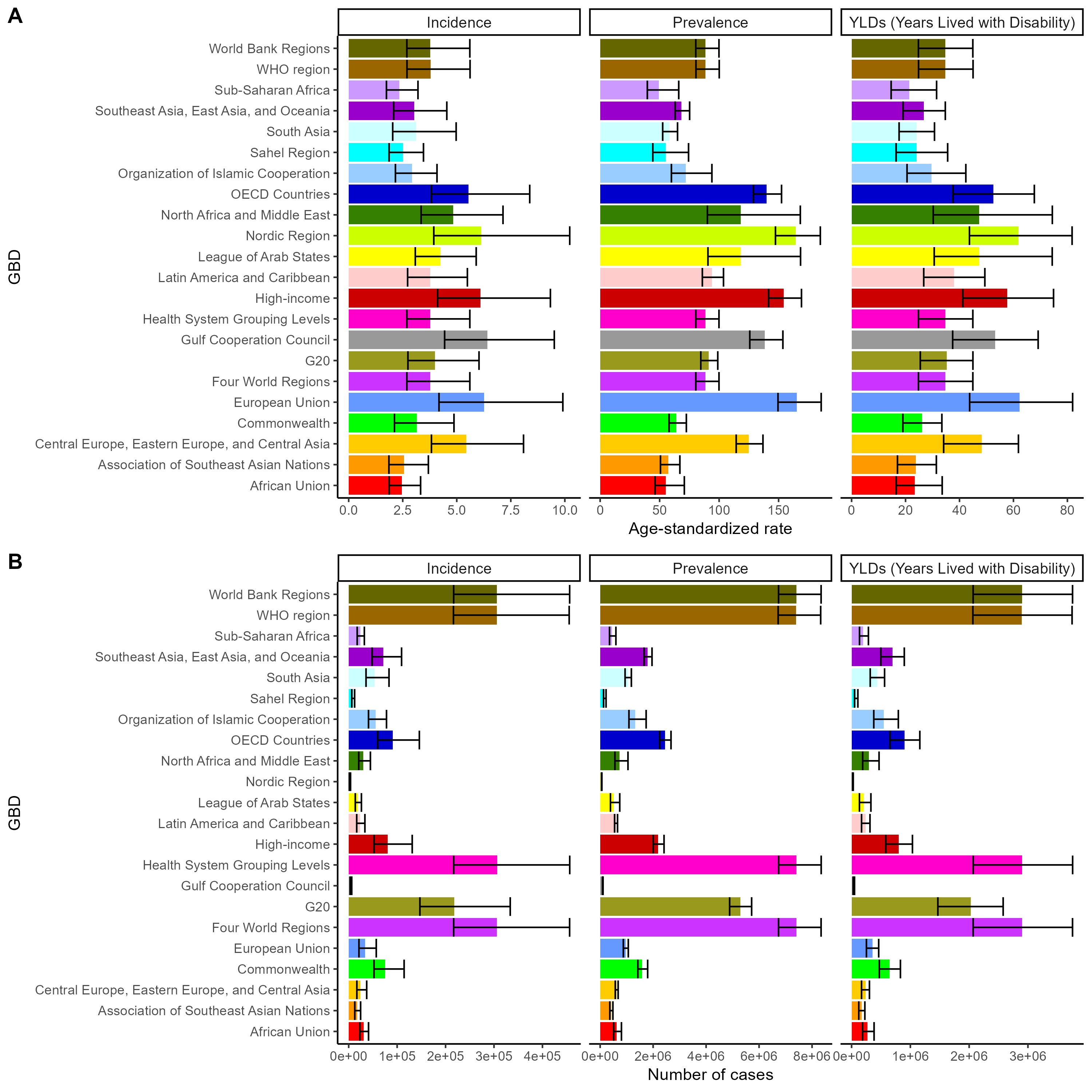

Supplement: Supplementary file 10 [file Figure_4.JPEG]

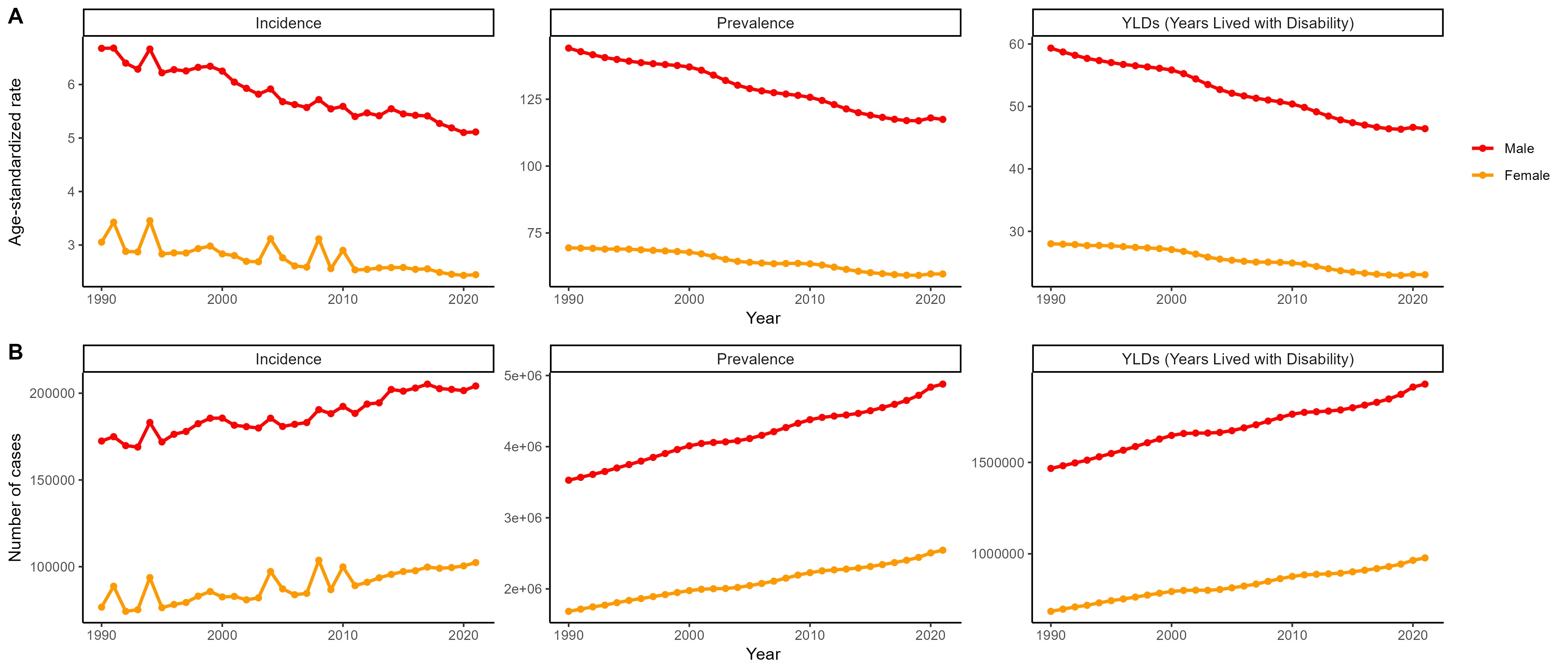

Supplement: Supplementary file 11 [file Figure_5.JPEG]

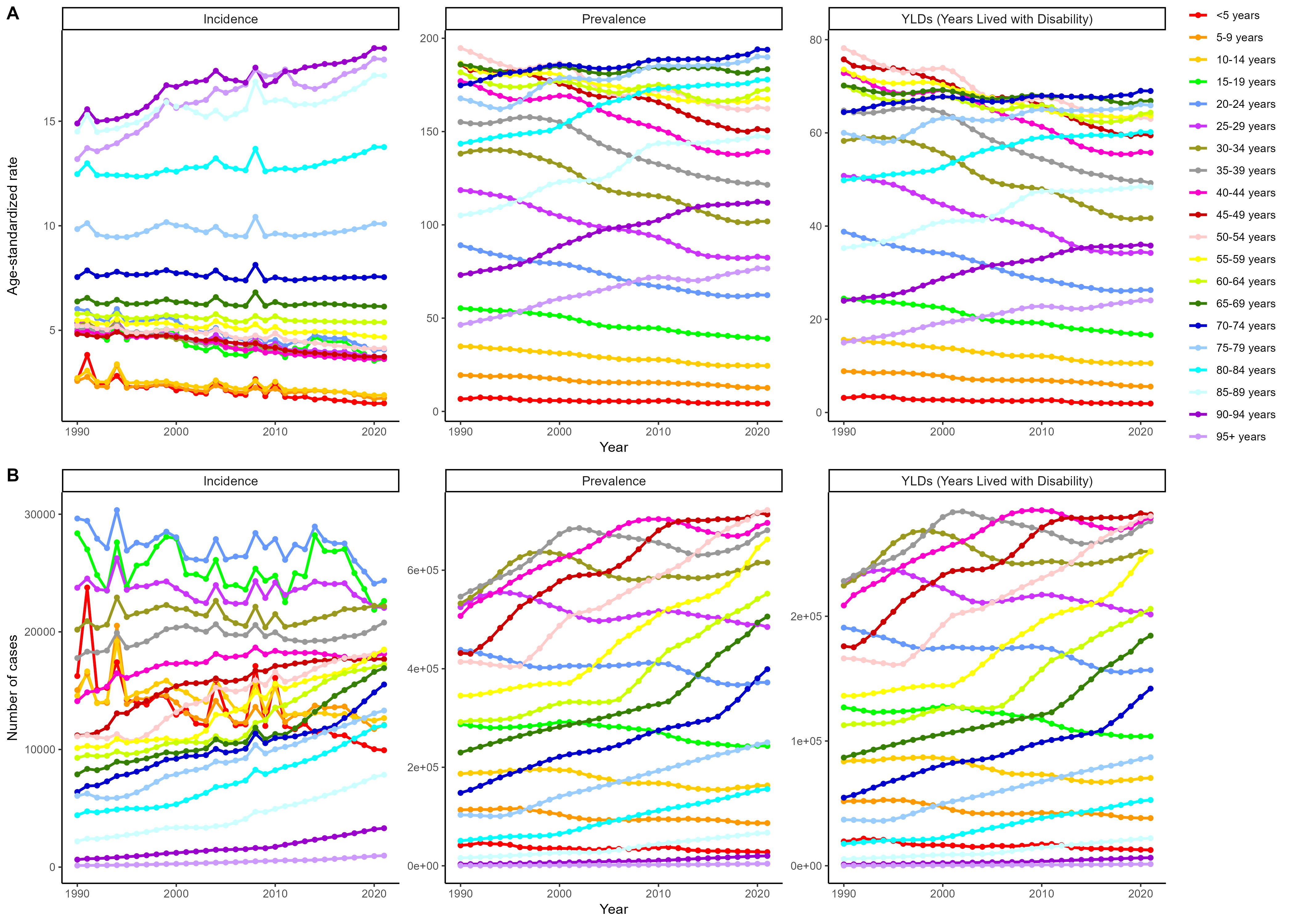

Supplement: Supplementary file 12 [file Figure_6.JPEG]

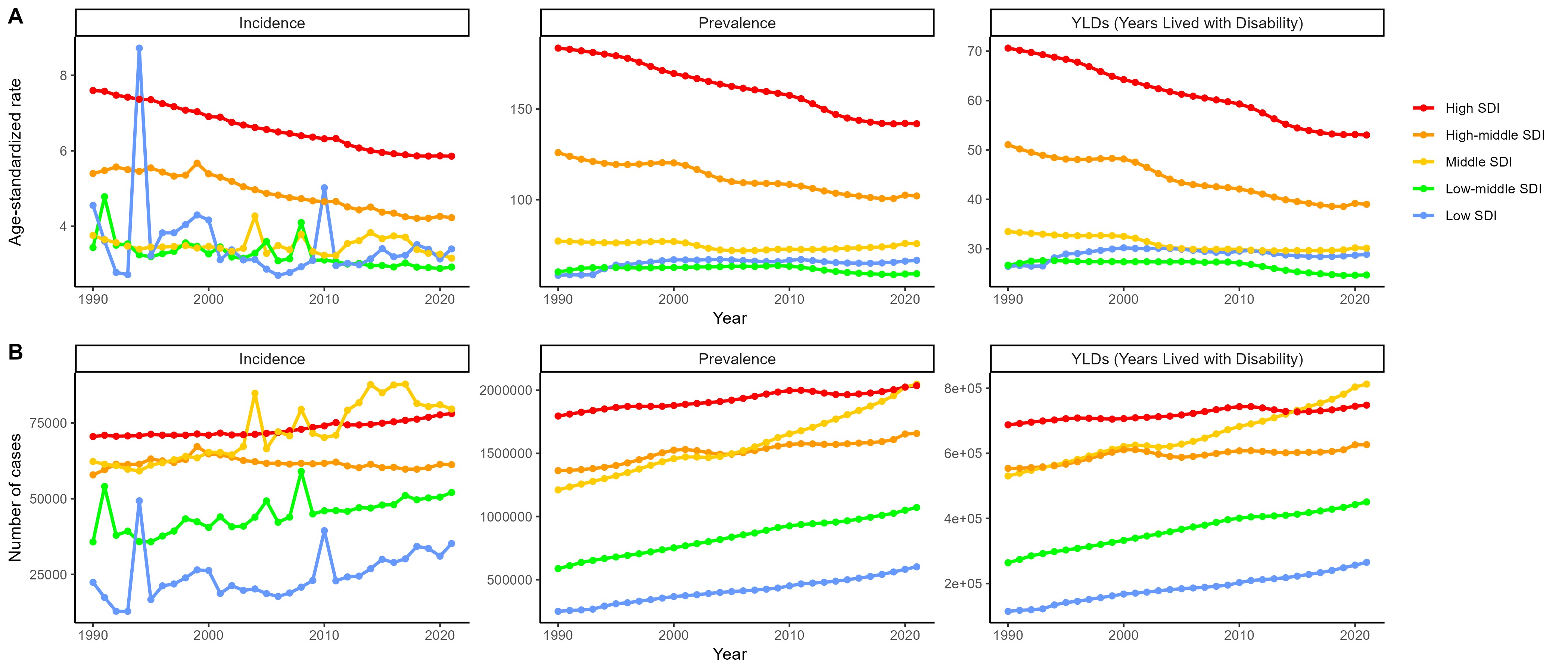

Supplement: Supplementary file 13 [file Figure_7.JPEG]

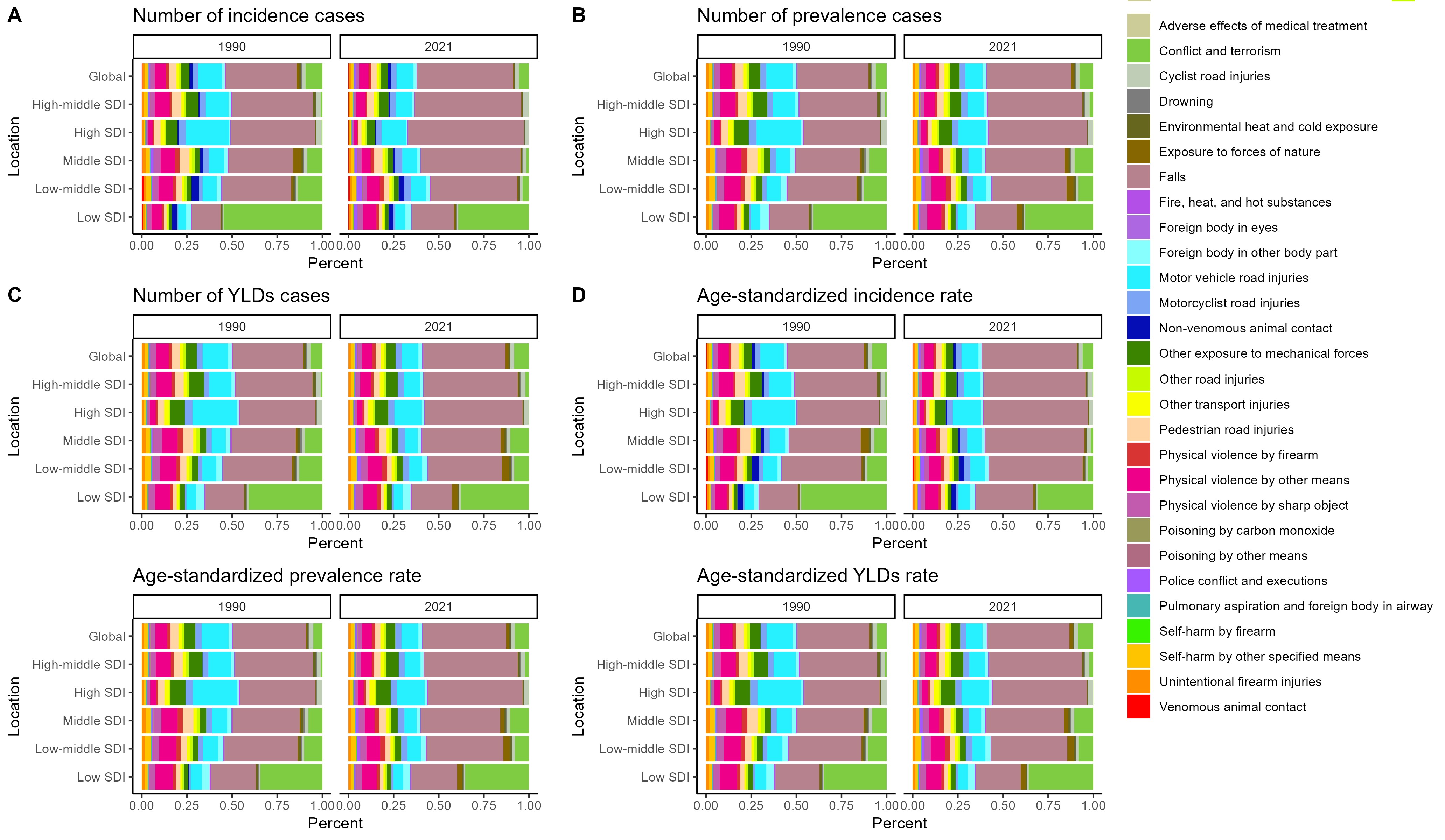

Supplement: Supplementary file 14 [file Figure_8.JPEG]
